# Half‐beam volumetric‐modulated arc therapy in adjuvant radiotherapy for gynecological cancers

**DOI:** 10.1002/acm2.13472

**Published:** 2021-11-16

**Authors:** Pei‐Chieh Yu, Ching‐Jung Wu, Hsin‐Hua Nien, Louis Tak Lui, Suzun Shaw, Yu‐Lun Tsai

**Affiliations:** ^1^ Department of Radiation Oncology Cathay General Hospital Taipei Taiwan; ^2^ School of Medicine China Medical University Taichung Taiwan; ^3^ Department of Radiation Oncology National Defense Medical Center Taipei Taiwan; ^4^ Department of Biomedical Engineering I‐Shou University Kaohsiung Taiwan; ^5^ School of Medicine Fu Jen Catholic University New Taipei City Taiwan; ^6^ Institute of Biomedical Engineering College of Electrical and Computer Engineering National Yang Ming Chiao Tung University Hsinchu Taiwan; ^7^ Oncology Treatment Center Sijhih Cathay General Hospital New Taipei City Taiwan; ^8^ Institute of Epidemiology and Preventive Medicine College of Public Health National Taiwan University Taipei Taiwan

**Keywords:** adjuvant radiotherapy, gynecological cancer, half‐beam volumetric‐modulated arc therapy

## Abstract

**Purpose:**

The purpose of this study is to introduce half‐beam volumetric‐modulated arc therapy (HVMAT), an innovative treatment planning technique from our work, for reducing dose to the organs at risk (OAR) during adjuvant radiotherapy for gynecological cancers.

**Methods and materials:**

Seventy‐two treatment plans of 36 patients with gynecological cancers receiving adjuvant radiotherapy were assessed. Among them, 36 plans were designed using HVMAT and paired with the other 36 traditional volumetric‐modulated arc therapy (VMAT) plans for each patient. The main uniqueness of the HVMAT designs was that it consisted of two opposite‐shielded half‐beam fields rotated inversely in two coplanar arcs, collocating with the specially‐devised avoidance structures to enhance the control of the OAR doses. The dose distributions in HVMAT and VMAT were evaluated and compared using the random effects model.

**Results:**

The ratios of OAR doses in HVMAT compared with VMAT showed a comprehensive OAR dose reduction when using HVMAT (*V*_20Gy_: bladder, 0.92; rectum, 0.95; *V*_30Gy_: bowel, 0.91; femoral heads, 0.66), except for the ilium (*V*_30Gy_: 1.12). The overall mean difference for each OAR across *V*_40Gy_, *V*_30Gy_, *V*_20Gy_, and bowel *V*_15Gy_ was statistically significant (almost all *p *< 0.001). In addition, HVMAT promoted a better conformity index, homogeneity index, *D*_2%_, and *V*_107%_ of the planning target volume (all *p *< 0.001).

**Conclusions:**

HVMAT is capable of generating deep double‐concave dose distributions with the advantage of reducing dose to several OARs simultaneously. It is highly recommended for pelvic irradiation, especially for treating gynecological cancers in adjuvant radiotherapy.

## 1 INTRODUCTION

Gynecological cancers cause a significant amount of morbidity and mortality worldwide, accounting for 10%–15% of all malignancies in women.^1^ Among them, both endometrial and cervical cancers are common. The incidence of endometrial cancer is high in developed countries, whereas cervical cancer is a leading cause of death in developing countries.^2^, ^3^

Adjuvant radiotherapy is an important modality for treating endometrial and cervical cancers.^4^ It is essential not only for patients with advanced diseases but also for those with early‐stage cancer to ensure long‐term survival.^5^, ^6^, ^7^, ^8^, ^9^, ^10^ Nevertheless, adverse events may also occur subsequently. Genitourinary and gastrointestinal complications are the most common grade ≥ 3 morbidities after pelvic irradiation.^11^, ^12^ To investigate the clinical outcomes and effects of treatment on late morbidity, the EMBRACE studies from the GEC‐ESTRO GYN working group and their relevant analyses offer reference materials for treatment parameters.^11^, ^12^, ^13^, ^14^, ^15^, ^16^ For organs close to the irradiated pelvic field, including the bladder, rectum, and bowel, they focus on the dose levels of 30 Gy and 40 Gy.^13^

In addition to genitourinary and gastrointestinal complications, pelvic bone complications are a serious issue attributable to pelvic irradiation that should also be considered.^17^, ^18^ Patients with gynecological cancer undergoing pelvic radiotherapy have increased risks of bone marrow toxicity and pelvic insufficiency fractures.^17^, ^18^ Treatment planning therefore becomes complicated because several OARs must be spared simultaneously. However, volumetric‐modulated arc therapy (VMAT) is generally associated with low‐dose bath to healthy tissues.^19^ This drives the motivation to develop a better planning technique to improve the disadvantages of the VMAT. The half‐beam volumetric modulated arc therapy (HVMAT) is therefore designed for gynecological cancers treated with adjuvant radiotherapy.

## 2 METHODS AND MATERIALS

### 2.1 Patients and target delineation

With the ethical approval of a local institutional review board, 36 patients with endometrial or cervical cancer receiving adjuvant radiotherapy at our institution were assessed. All patients had a disease status ranging from stages IA to IIIC and had undergone a radical surgery that included a total hysterectomy with only the vaginal cuff left. The treatment target was delineated based on images of computed tomography (CT) simulation with a full bladder set up by drinking 300– 400 ml water 30–40 min prior to the scan. The clinical target volume (CTV) definitions were set according to the NRG oncology/RTOG consensus guidelines, including 3.5–4.0 cm of the proximal vagina as well as paravaginal and retracted parametrical tissue with considerations for internal organ motion; the common, external, and internal iliac nodal regions with a 0.7 cm margin around the vessels; and the obturator nodal region. For patients with cervical cancer, or endometrial cancer with cervical stromal invasion or known lymph node metastases, the presacral nodal region was also involved.^20^ The planning target volume (PTV) was generated by expanding 0.7 cm from the CTV in all directions.^20^

### 2.2 HVMAT and VMAT planning

**FIGURE 1:**
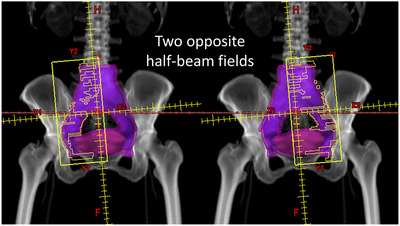
HVMAT consists of two opposite‐shielded half‐beam fields within two inverse fully‐rotated coplanar arcs. One rotates clockwise (181° to 179°) with the left side shielded. The other one rotates counterclockwise (178° to 182°) with the right side shielded. Abbreviation: HVMAT, half‐beam volumetric‐modulated arc therapy

The prescribed dose was 45.0 Gy in 1.8 Gy per fraction to the PTV. A total of 72 treatment plans comprising 36 HVMAT plans paired with the 36 VMAT plans of each patient were made. All plans were designed in two full coplanar arcs, with one rotating clockwise (181° to 179°) and the other one rotating counterclockwise (178° to 182°). For HVMAT, both arcs contained one half‐beam field with the left or right side shielded, respectively. Figure 1 shows the design of the two opposite‐shielded half‐beam fields used in HVMAT planning. For VMAT, the arc rotations were the same as for HVMAT, but the fields were unshielded. Two arcs were optimized simultaneously in both types of planning.

The treatment plans were designed using the Eclipse™ treatment planning system (TPS), version 11.5 (Varian Medical Systems, Palo Alto, CA, USA), for the linear accelerator, which was the Truebeam STx (Varian Medical Systems, Palo Alto, CA, USA) equipped with a high‐definition 120‐leaf multileaf collimator (MLC) with 2.5 mm leaves in the middle and 5 mm leaves on the sides. The maximum dose rate was set to 400 MU/min with 10 MV photon beams. The grid size for dose calculation was 2.5 mm. The anisotropic analytical algorithm, version 11.0.31, was used for volume dose calculation, and the progressive resolution optimizer, version 11.0.31, was used for planning optimization.

### 2.3 Planning optimization

**FIGURE 2:**
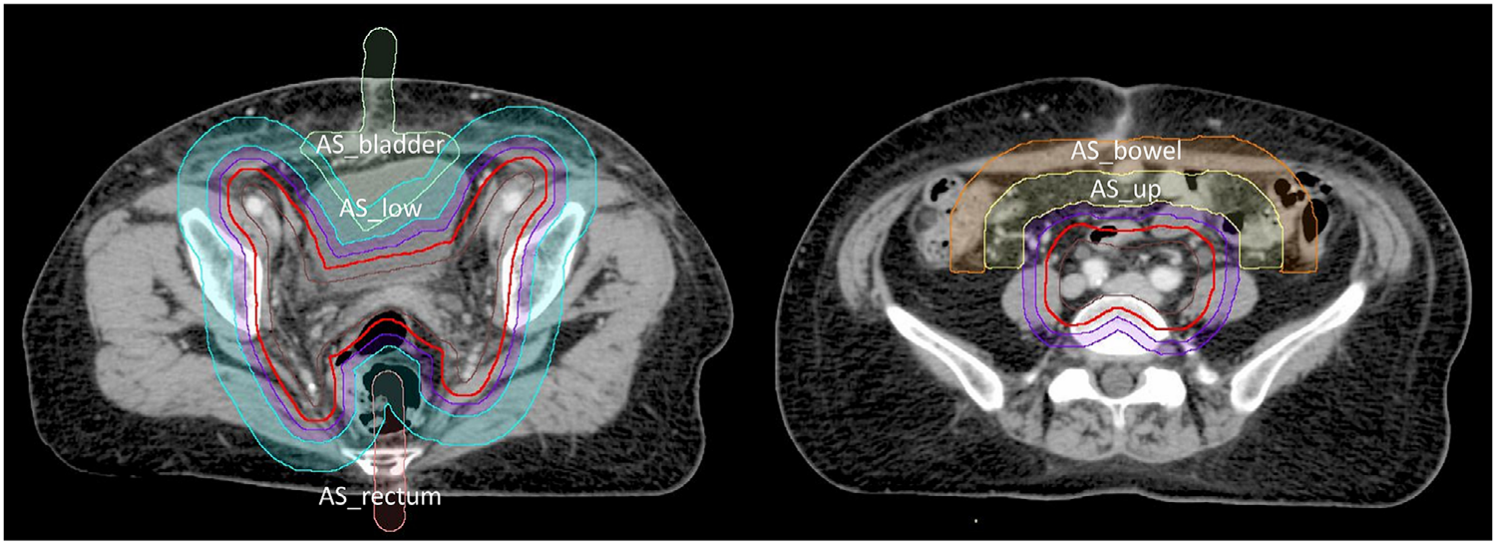
Avoidance structures for planning optimization in addition to normal OAR. A 0.5‐cm wide ring (purple) surrounds the entire PTV (red) with a distance of 0.5 cm. Another two 1.5‐cm wide structures, named AS_low (cyan, ring shape) and AS_up (yellow, rainbow shape), surround the ring to strengthen the dose falloff and are separated by the superior border of the sacroiliac joint. Three individual 2‐cm‐wide structures are designed for avoiding dose spillage to the bladder, rectum, and bowel, named AS_bladder (green, arrow shape), AS_rectum (pink, stick shape), and AS_bowel (orange, rainbow shape), respectively. Abbreviations: OAR, organs at risk; PTV, planning target volume

In addition to the normal organs of bladder, rectum, bowel bag, ilium, femoral heads, and spinal cord, six avoidance structures were designed to enhance the control of the doses to organs at risk (OAR). Figure 2 illustrates the delineation of these six avoidance structures for planning optimization. They include a 0.5‐cm wide ring structure to primarily increase the dose falloff, and another two 1.5‐cm wide structures separated by the superior border of the sacroiliac joint and surrounding the ring to further strengthen the dose falloff. The other three 2‐cm wide structures with specific shapes were individually designed for avoiding dose spillage to the bladder, rectum, and bowel. Among them, the structures for the bladder and rectum extended beyond the body surface to prevent radiation beams from the contralateral side. All structures were at least 0.5‐cm away from the PTV to preclude dose conflicts between the target and avoidance structures during planning optimization.

Table 1 summarizes the dose–volume constraints and priorities for planning optimization regarding all structures. This objective template was applied in every plan with no discrepancies to avoid systematic biases between different types of planning during optimization. The template was used for the whole optimization process (multi‐resolution 1–4) followed by an additional optimization (multi‐resolution 4 only). The main scheme was to achieve at least the *D*_95%_ of PTV ≥ 42.5 Gy, which was 95% of the prescribed dose, while keeping the bladder *V*_45Gy_ ≤ 35_%_, the rectum *V*_30Gy_ ≤ 60%, and the bowel *V*_40Gy_ ≤ 30_%_.^21^ An experienced medical physicist made all plans with no individual adjustments for different patients.

**TABLE 1:** Dose–volume parameters and priorities for planning optimization

**Structure**	**Volume (%)**	**Dose (cGy)**	**Priority**	**Structure**	**Volume (%)**	**Dose (cGy)**	**Priority**	**Structure**	**Volume (%)**	**Dose (cGy)**	**Priority**
PTV	0	4623	800	CTV	100	4610	600				
	15	4622	600		99	4611	600				
	100	4580	600		98	4612	600				
	98	4581	600								
Bladder	40	2264	500	Rectum	43	1635	500	Bowel	8	2955	500
	27	2498	500		30	2062	500		4	3141	500
	15	2799	500		15	2526	500		2	3330	500
	3	3246	500		2	3011	500		0	3514	500
Spinal cord	15	2847	300	Ilium	80	470	200	Femoral head	23	1194	50
	6	2895	300		64	1100	200		3	1950	50
	0	2968	300								
AS_bladder	26	1113	400	AS_rectum	34	1465	400	AS_bowel	23	1709	400
	13	1339	400		20	1607	400		16	1782	400
	2	1698	400		11	1714	400		9	1881	400
					2	1753	400		4	2015	400
									0	2062	400
Ring	42	1697	100	AS_low	38	2474	100	AS_up	31	2270	300
	31	2214	100		24	2778	100		16	2465	300
	19	2883	100		11	3095	100		3	2648	300
	10	3342	100		1	3544	100				
	3	4080	100								

*Abbreviations*: AS, avoidance structure; CTV, clinical target volume; PTV, planning target volume.

### 2.4 Plan evaluation

The goodness of the HVMAT and VMAT plans was determined primarily by the dose distributions of OAR, including the bladder, rectum, bowel, ilium, and femoral heads. The representative dose–volume parameters were *V*_40Gy_, *V*_30Gy_, and *V*_20Gy_. For the bowel, *V*_15Gy_ was additionally evaluated because of its importance regarding grade ≥ 3 toxicities.^22^ Body *V*_20Gy_ was the parameter used for the comparison of the low‐dose bath between these two techniques. Furthermore, to understand the benchmark of the target dose at which the OAR doses were analyzed, the conformity index (CI), homogeneity index (HI), *D*_2%_, and *V*_107%_ of PTV were assessed as the second endpoint.^13^, ^23^, ^24^

### 2.5 Statistics

The comparisons of OAR and PTV doses between HVMAT and VMAT plans were conducted using the random effects model with random intercepts, which can estimate the mean difference between HVMAT and VMAT and its *p* value across different dose–volume parameters, with adjustments for different patients under the assumption of independent and identically distributed random variables.^25^, ^26^ The statistical calculations were applied in R, version 4.0.4, a programming language and software environment for statistical computing and graphics supported by the R Foundation for Statistical Computing. A *p* value < 0.05 was considered significant.

## 3 RESULTS

### 3.1 OAR dose evaluation

**FIGURE 3:**
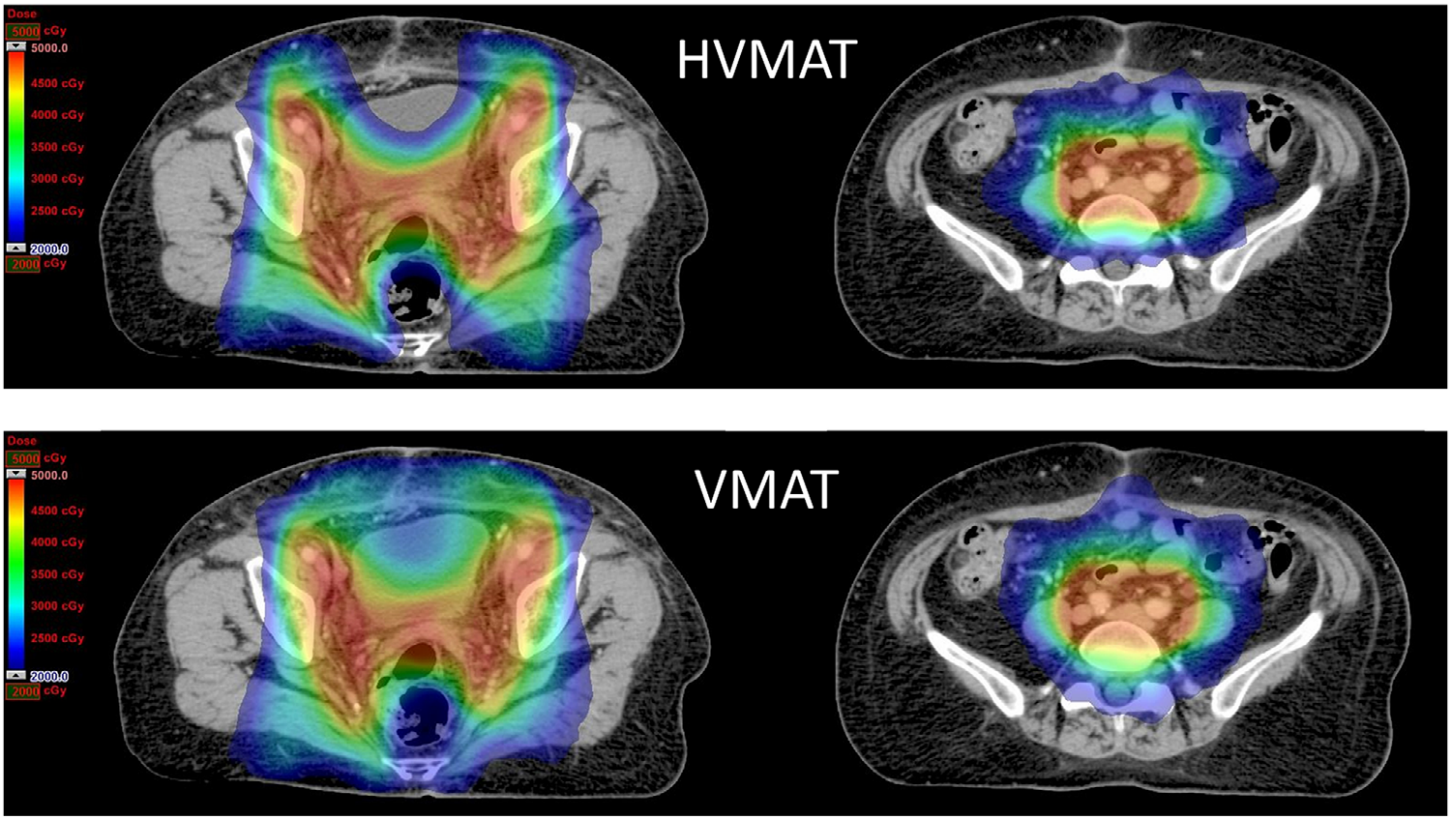
Dose distributions of HVMAT and VMAT at the bladder, rectum, and bowel levels. Abbreviations: HVMAT, half‐beam volumetric‐modulated arc therapy; VMAT, volumetric‐modulated arc therapy

Figure 3 indicates that HVMAT facilitated more concentrated dose distributions, which reduced dose to the OAR. Table 2 demonstrates the result of a comprehensive OAR dose reduction in HVMAT compared with VMAT at the expense of a 10% higher ilium dose at *V*_30Gy_ and *V*_40Gy_. The lowest mean ratio of the doses in HVMAT against VMAT for each OAR appeared at *V*_20Gy_ of the bladder (0.92) and rectum (0.95), and *V*_30Gy_ of the bowel (0.91) and femoral heads (0.66). The overall mean differences for the OAR across *V*_40Gy_, *V*_30Gy_, *V*_20Gy_, and bowel *V*_15Gy_ were statistically significant (almost all *p *< 0.001). Body *V*_20Gy_ also showed a significant difference between HVMAT and VMAT (*p *< 0.001), with a mean ratio of 0.97, which indicates that HVMAT can reduce low‐dose bath to the patients. For a single type of cancer, both endometrial and cervical cancers demonstrated similar results compared to the pooled analysis.

**TABLE 2:** OAR dose evaluation for both types of gynecological cancer

		**HVMAT**	**VMAT**			
		**mean (SD)**	**mean (SD)**	**HVMAT/VMAT mean ratio**	**HVMAT‐VMAT mean difference**	***p*‐Value**
Bladder	*V*_20Gy_ (%)	74.3 (7.5)	80.9 (7.6)	0.92		
	*V*_30Gy_ (%)	51.8 (9.5)	55.2 (8.5)	0.93		
	*V*_40Gy_ (%)	34.7 (10.5)	34.3 (9.9)	1.01		
	Overall difference (%)				−3.21	<0.001*
Rectum	*V*_20Gy_ (%)	67.9 (9.1)	71.2 (9.0)	0.95		
	*V*_30Gy_ (%)	46.3 (11.3)	47.5 (10.9)	0.97		
	*V*_40Gy_ (%)	25.9 (10.5)	24.8 (10.6)	1.05		
	Overall difference (%)				−1.12	0.025*
Bowel	*V*_15Gy_ (%)	78.2 (12.4)	80.3 (13.2)	0.97		
	*V*_20Gy_ (%)	60.3 (12.4)	63.8 (13.8)	0.94		
	*V*_30Gy_ (%)	31.0 (9.3)	34.4 (11.3)	0.91		
	*V*_40Gy_ (%)	14.0 (5.8)	14.9 (6.2)	0.93		
	Overall difference (%)				−2.49	<0.001*
Ilium	*V*_20Gy_ (%)	63.4 (8.3)	63.6 (9.8)	1.00		
	*V*_30Gy_ (%)	34.5 (8.2)	30.9 (7.7)	1.12		
	*V*_40Gy_ (%)	16.8 (5.7)	14.9 (4.5)	1.11		
	Overall difference (%)				1.72	0.001*
Femoral heads	*V*_20Gy_ (%)	13.5 (5.3)	19.0 (5.1)	0.71		
	*V*_30Gy_ (%)	3.5 (2.7)	5.0 (2.4)	0.66		
	*V*_40Gy_ (%)	0.6 (1.1)	0.7 (0.9)	0.78		
	Overall difference (%)				−2.35	<0.001*
Body	*V*_20Gy_ (%)	17.4 (3.1)	17.7 (3.0)	0.97	−0.38	<0.001*

*Notes*

* Statistical significance.

*Abbreviations*: HVMAT, half‐beam volumetric modulated arc therapy; SD, standard deviation; VMAT, volumetric‐modulated arc therapy.

### 3.2 PTV dose evaluation

Table 3 denotes that HVMAT promoted a better target dose. All the CI, HI, *D*_2%_, and *V*_107%_ of PTV in HVMAT were superior to those in VMAT with statistical significance (all *p *< 0.001), meaning that the PTV dose was more conformal (CI: 0.83 vs. 0.82), more homogeneous (HI: 0.10 vs. 0.14), and with fewer hot spots (*D*_2%_: 48.6 Gy vs. 49.1 Gy; *V*_107%_: 126.6 ml vs. 187.9 ml) when using HVMAT.

**TABLE 3:** PTV dose evaluation for both types of gynecological cancer

		**HVMAT**	**VMAT**	
		**mean (SD)**	**mean (SD)**	***p*‐Value**
PTV	CI	0.83 (0.02)	0.82 (0.02)	<0.001^*^
	HI	0.10 (0.02)	0.14 (0.03)	<0.001^*^
	*D*_2%_ (Gy) *V*_107%_ (ml)	48.6 (0.19) 126.6 (52.9)	49.1 (0.34) 187.9 (61.7)	<0.001^*^ < 0.001^*^

*Abbreviations*: CI, conformity index; HI, homogeneity index; HVMAT, half‐beam volumetric‐modulated arc therapy; PTV, planning target volume; SD, standard deviation; VMAT, volumetric‐modulated arc therapy.

### 3.3 Plan delivery

Under the 3%/3 mm criterion of gamma analysis, portal dosimetry was carried out and passed for all HVMAT plans. On average, the passing rate of area gamma < 1 was 98.9% with a tolerance limit of 97%, the maximum gamma was 1.9 with the tolerance limit of 3, and the average gamma was 0.3 with the tolerance limit of 0.5. The average monitor unit (MU) of HVMAT was 827.8 MU delivered in 2.1 min, which was more than that of VMAT, with an average of 582.6 MU delivered in 2.0 min.

## 4 DISCUSSION

The comprehensive dosimetric advantages of HVMAT are derived from several tailored designs that confine the dose to PTV with less dose to the OAR. The designs involve the half‐beam fields, the avoidance structures, and the arcs used in HVMAT to generate deep double‐concave dose distributions for preserving several OAR simultaneously.

Half‐beam fields are the most essential part of generating double‐concave dose distributions. Each field focuses only on one hemi‐side of the body at a time to sculpt the dose to fit PTV and to shield the other hemi‐side of the body using jaws, meaning that the leaves of the MLC do not need to be moved back and forth across the midline frequently, which can facilitate better shielding of the OAR. This advantage can be observed in the difference in the average MU of HVMAT and VMAT. The average MU of HVMAT is 1.4 times more than that of VMAT, which means that the selection of MU is more delicate in HVMAT. Former studies showed that permanent shielding is beneficial in creating mono‐concave dose distributions for anal cancers.^27^ Other researchers found two arcs optimal.^28^, ^29^ In our HVMAT design, the innovation of two permanent opposite‐shielded half‐beam fields creates double‐concave dose distributions that fit better for the geometry of PTV in gynecological cancers. Moreover, half beams reduce beam divergence that may also help beam focusing.

The avoidance structures also contribute much to the double‐concave dose distributions. A single anterior avoidance structure was formerly proven to be a useful tool when planning rectal and anal canal cases.^30^ For the more complicated dose distributions that gynecological cancers require, we apply three individual structures with special shapes to control the dose received by the bladder, rectum, and bowel respectively, with no dose conflicts between one another. In addition, structures extend outside the body, reducing dose from the contralateral side. The avoidance structures work well with the half‐beam fields to achieve deep double‐concave dose distributions.

The bladder, rectum, and bowel are the ordinary OAR in pelvic radiotherapy. However, the important dose–volume parameters for each of them are not yet conclusive. In general, the dose levels being cared about for the bladder and rectum are usually higher than the dose levels for the bowel.^31^, ^32^, ^33^, ^34^, ^35^, ^36^, ^37^ The range of dose levels in regard to grade ≥ 2 urinary incontinence, grade ≥ 1 hematuria, and grade ≥ 1 pain during urination is from 40 Gy to 75 Gy.^31^ For grade ≥ 2 late rectal toxicity and grade ≥ 1 rectal bleeding, the range is between 21 Gy and 73 Gy.^32^ On the other hand, concerning bowel toxicity such as grade ≥ 2 diarrhea and grade ≥ 2 enteritis, the range is as low as 5 Gy but as high as 50 Gy in some literature.^33^, ^34^, ^35^, ^36^, ^37^ Although the prescribed dose only goes up to 45 Gy in our study, it is dangerous if the care of low‐dose bath, which is a general drawback of VMAT, is not taken during treatment planning and plan evaluation. HVMAT is therefore a valuable planning technique because it intrinsically reduces OAR doses by means of its designs.

The ilium and femoral heads are complementary normal structures during planning optimization, and the doses are evaluated in our study. Bone complications are not uncommon after pelvic radiotherapy. The proportion of patients with radiation‐induced pelvic bone and femoral head complications is approximately 31.1%, including 13.9% with pelvic insufficiency fracture, 12.3% with red bone marrow changes, 4.1% with radiation osteitis, and 0.8% with avascular necrosis of the femoral head.^38^ The median time from the end of radiotherapy to the diagnosis of bone complications is around 25 months.^38^ However, the doses can be decreased if attention is paid to them. Using the bone structures during planning, optimization effectively reduces the doses without increasing the doses to the bladder, rectum, and bowel.^39^ HVMAT can be applied properly with care and attention to detail.

Using daily image guidance such as daily cone beam CT is strongly recommended in combination with HVMAT plans. One reason is because of the tight dose distributions generated by HVMAT. The other is because of the tight vaginal PTV margin used in our study. A vaginal PTV margin of 0.6–0.8 cm can be safe if considering internal organ motion and applying daily cone beam CT image guidance to ensure accurate setup and bladder fill and rectal distension.^20^ A margin of 1.5–2.0 cm or greater is recommended if no or minimal image guidance is employed.^20^

The use of HVMAT in other tumor groups, such as gynecological tumor without surgical resection and prostate cancer requiring pelvic nodal irradiation, is feasible. Double‐concave dose distributions are also needed in these clinical scenarios. Although the OAR doses reduced in a definitive setting are not as much as in an adjuvant setting because the tumor exists, HVMAT still demonstrated dosimetric benefits in our additional examination. Seminal vesicle irradiation is another issue in treating prostate cancer. The elongation of seminal vesicles laterally and superiorly from the prostate between the bladder and rectum can cause a significant dose increase around this area, especially because the dose prescribed for prostate cancer is usually higher. Hence, despite not being the main subject in this study, using HVMAT in other tumor groups has potential.

This study has some limitations. First, it was a planning comparison study. Although the results were clear and statistically significant, it only yielded logical advantages of HVMAT over VMAT in dosimetric data. Radiotherapy is carried out for better clinical outcomes of patients rather than merely for better treatment planning parameters. Therefore, further clinical follow‐up is essential to examine whether the dosimetric advantages can be finally translated into clinical significance, particularly for those with small differences between HVMAT and VMAT. Second, the half‐beam design requires the TPS supporting manually opened half‐beam fields with at least fixing the jaw at the side of permanent shielding. For the TPS that the jaw opens automatically according to the target, the half‐beam design could not be achieved successfully. Third, the extension of avoidance structures exceeding the body surface is a strategy limited to certain planning algorithms. For those with the optimization and calculation volume only within the patient body, this strategy may be unnecessary. Fourth, our study only focused on patients needing pelvic irradiation. If para‐aortic irradiation is also demanded, the duodenum, kidneys, and possible transposed ovaries are also OAR abutting the irradiated volume.^13^ In this case, the ability of HVMAT to avoid dose spillage anteriorly to the bowel may decrease because these OAR contribute much dose restriction from the lateral side. More attention must be paid to bowel toxicity in this situation.

## 5 CONCLUSIONS

HVMAT is an innovative treatment planning technique designed for gynecological radiotherapy. By means of its crafted designs involving the two opposite‐shielded half‐beam fields and the specially‐devised avoidance structures, it is capable of generating deep double‐concave dose distributions with the advantages of reducing dose to the OAR comprehensively also facilitating a better PTV dose. In clinical applications, it is highly recommended for treating gynecological cancers in adjuvant radiotherapy that requires the preservation of several nearby OAR simultaneously.

## CONFLICT OF INTEREST

The authors declare that there is no conflict of interest that could be perceived as prejudicing the impartiality of the research reported.

## AUTHORS’ CONTRIBUTIONS

PCY participated in the design of the study, data collection, and paper writing. CJW involved in the acquisition of data and patient care. HHN conducted the research project. LTL involved in the acquisition of data and patient care. SS involved in the acquisition of data and patient care. YLT participated in the revising of the manuscript critically for important intellectual content. All authors read and approved the final manuscript.

## Supporting information

Supporting Information
