# Supplementary material for: Half‐beam volumetric‐modulated arc therapy in adjuvant radiotherapy for gynecological cancers
Source: J Appl Clin Med Phys. 2021 Nov 16;23(1):e13472. doi: 10.1002/acm2.13472 (PMC8803303; doi:10.1002/acm2.13472)
Supplement: Supplementary file 1 — Supporting Information [file ACM2-23-e13472-s001.docx]

**Half-beam Volumetric Modulated Arc Therapy in Adjuvant Radiotherapy for Gynecological Cancers**

Running title: HVMAT for gynecological cancers

Pei-Chieh Yu, Ph.D.^1,2^, Ching-Jung Wu, M.D.^1,3,4^, Hsin-Hua Nien, M.D.^1,5,6^, Louis Tak Lui, M.D.^1^, Suzun Shaw, M.D.^7^, and Yu-Lun Tsai, M.D.^1,8,*^

1. Department of Radiation Oncology, Cathay General Hospital, Taipei, Taiwan
2. School of Medicine, China Medical University, Taichung, Taiwan
3. Department of Radiation Oncology, National Defense Medical Center, Taipei, Taiwan
4. Department of Biomedical Engineering, I-Shou University, Kaohsiung, Taiwan
5. School of Medicine, Fu Jen Catholic University, New Taipei City, Taiwan
6. Institute of Biomedical Engineering, College of Electrical and Computer Engineering, National Yang Ming Chiao Tung University, Hsinchu, Taiwan
7. Oncology Treatment Center, Sijhih Cathay General Hospital, New Taipei City, Taiwan
8. Institute of Epidemiology and Preventive Medicine, College of Public Health, National Taiwan University, Taipei, Taiwan

Corresponding author: Yu-Lun Tsai, M.D., Department of Radiation Oncology, Cathay General Hospital, 280 Renai Rd. Sec.4, Taipei, Taiwan

Tel: +886227082121#3711; E-mail: tuna0305@hotmail.com.tw

Pei-Chieh Yu: [janeyu115@gmail.com](mailto:janeyu115@gmail.com), Ching-Jung Wu: [cgh01222@cgh.org.tw](mailto:cgh01222@cgh.org.tw),

Hsin-Hua Nien: [cgh10370@cgh.org.tw](mailto:cgh10370@cgh.org.tw), Louis Tak Lui: [louistlui@yahoo.com](mailto:louistlui@yahoo.com),

Suzun Shaw: [cgh00422@cgh.org.tw](mailto:cgh00422@cgh.org.tw), Yu-Lun Tsai: tuna0305@hotmail.com.tw

All authors have no conflicts of interest to disclose.

The datasets used and analyzed during the current study are available from the corresponding author on reasonable request.

The present study is ethically approved by institutional review board of the Cathay General Hospital. The reference number is CGH-P108083.

The research was conducted in the Department of Radiation Oncology, Cathay General Hospital, 280 Renai Rd. Sec.4, Taipei, Taiwan.

**Acknowledgments**

This work was supported by the Cathay General Hospital institutional grant (CGH-MR-B10914).

**Authors’ contributions**

PCY participated in the design of the study, data collection, and paper writing. CJW involved in the acquisition of data and patient care. HHN conducted the research project. LTL involved in the acquisition of data and patient care. SS involved in the acquisition of data and patient care. YLT participated in the revising of the manuscript critically for important intellectual content. All authors read and approved the final manuscript.
